# Impairment in Preattentive Processing among Patients with Hypertension Revealed by Visual Mismatch Negativity

**DOI:** 10.1155/2014/945121

**Published:** 2014-03-26

**Authors:** Cuiping Si, Changjie Ren, Peng Wang, Hetao Bian, Haiming Wang, Zhongrui Yan

**Affiliations:** ^1^Department of Neurology, Affiliated Jining First People's Hospital, Shandong Academy of Medical Sciences, No. 6, Jiankang Road, Jining, Shandong 272011, China; ^2^School of Medicine and Life Science, University of Jinan-Shandong Academy of Medical Sciences, Jinan 250000, China

## Abstract

*Objective.* Patients with hypertension show deficits in cognitive function. However, the neural mechanisms underlying the preattentive information processing in hypertensive patients are poorly understood. We seek to investigate whether hypertensive patients have impairments in preattentive information processing. *Methods.* We compared visual mismatch negativity (vMMN) between 15 hypertensive patients and 15 age-matched healthy controls, which was elicited by the change of visual duration randomly presented in both peripheral visual fields. In addition, the global cognitive function for all participants was assessed with Mini-Mental State Examination (MMSE). *Results.* The vMMN in deviant-standard comparison was observed at occipital-temporal regions. Compared with normal healthy controls, the amplitude of vMMN was significantly decreased in hypertensive patients (*P* < 0.05). Meanwhile, the vMMN peak latency was delayed in the hypertensive group (*P* < 0.05). However, the MMSE scores of patients with hypertension were not significantly different from those of controls (*P* > 0.05), and there was no significant correlation between the mean amplitude of vMMN and SBP, DBP, and MMSE in hypertensive individuals, respectively. *Conclusions.* These data indicate dysfunction of automatically change detection processing in patients with hypertension. Moreover, the changes of vMMN provide a more objective and reliable assessment for cognitive impairment in hypertensive patients.

## 1. Introduction

Hypertension, as one of the most common chronic diseases among adults, contributes to the development of various cardiovascular [1] and neurological disorders such as stroke [2], Alzheimer's disease [3–5], and vascular dementia [3, 6]. Moreover, patients with hypertension showed cognitive dysfunction in several domains such as executive function, memory, calculation, and orientation [6–11]. In addition, recent studies suggest that attention deficits are also associated with hypertension [7, 8, 12]. Preattentive information processing is an essential cognitive function for humans that occurs at the very early stage of information processing [13]. Detecting changes in the surrounding environment outside of conscious attention plays an important role in survival [14]. Particularly, visual preattentive change detection is both possible and necessary for the human visual system to quickly and effectively detect sudden changes in the absence of attention [15]. However, whether preattentive processing is impaired in hypertensive patients is still unclear.

The mismatch negativity (MMN) is the reliable indicator for exploring preattentive processing, reflecting the brain ability to automatically extract task-irrelevant information under nonattentional conditions as well as indexes of preattentive sensory memory information processing [16, 17]. It is defined by a negative deflection during the time range of approximately 100–250 ms after stimulus onset, and this response is elicited by infrequent stimuli (deviant) compared to repeated stimuli (standard) [16]. While the MMN component being well defined in the auditory modality, visual MMN (vMMN) has been widely found for deviant visual information such as color [18, 19], shape [20], size [21], luminance [22], orientation [23], and duration [24]. Noteworthily, it has been shown that the vMMN amplitude was changed in various diseases. For instance, the amplitude of vMMN was decreased in patients with schizophrenia [25] and a major depressive disorder [24] but increased in Alzheimer's disease [26], suggesting an impairment of preattentive information processing in these patients.

There were overwhelming majorities of studies that showed a negative correlation between hypertension and cognitive function [6–11, 27–29]. Particularly, the N2 and P3 components of ERPs were shown to be decreased in hypertensive patients compared to normal controls, suggesting the existence of early changes in neurocognitive function in early hypertension [30, 31]. Based on these findings, hypertensive patients may exhibit dysfunction of preattentive change detection; MMN should be smaller compared to normal controls. So we aim to examine preattentive processing in hypertensive patients by recording the mismatch negativity (vMMN) of visual duration changes.

In this study, we determined whether information processing at the preattentive stage is functionally impaired in hypertensive patients by recording the duration of MMN using the deviant-standard paradigm. Under this paradigm, vMMN can be recorded with low-probability (20%) duration deviant stimuli (50 ms) inserted randomly in a sequence of frequent (80%) standard stimuli (100 ms). In the center of screen, a black cross was displayed throughout the stimulus blocks. From time to time, the cross became bigger or smaller unpredictably. In order to effectively control the attention, participants were instructed to ignore the peripheral stimuli and press the left or the right button as quickly and accurately as possible when the size of the cross became bigger or smaller [20]. Compared to the standard stimuli, deviant stimulus elicited clearly negative detection between 150 and 300 ms after stimulus [32].

## 2. Methods

### 2.1. Participants

According to WHO Guidelines (2003), hypertension was defined as the mean of two readings of systolic blood pressure (SBP) of 140 mmHg or greater, diastolic BP (DBP) of 90 mmHg or greater, or having a self-report history of hypertension [33]. Then, we selected 15 hypertensive patients from Jining First People's Hospital, and 15 normotensive individuals that were matched with the patients on age, gender, and education were recruited as controls. All participants who were diagnosed as having other affective disorders such as secondary hypertension, stroke, Alzheimer's disease, mental illness, drug dependence, alcohol dependence, and diabetes mellitus were excluded. Global cognitive function was assessed with the Mini-Mental State Examination (MMSE). The MMSE is a test of orientation, attention, calculation, language, and recall with a score of 0–30; less than or equal to 24 shows significant decline in cognitive function. All subjects were right-handed, with normal or corrected-to-normal vision. This study was approved by the Ethics Committee at the Jining First People's Hospital in Shandong, China. A written informed consent was obtained from all participants before the experiment.

### 2.2. Stimuli and Procedure

Two identical visual stimuli were simultaneously presented in peripheral sides of the field with a visual angle of 4.5° × 4.5° from a distance of 1 m as previously reported [32]. The stimulus onset asynchrony (SOA) was fixed at 600 ms. In the present study, the solid red rectangles (30 mm in length and 10 mm in width) were as standard stimuli (probability 80%), with the presentation of 50 ms. Meanwhile, there were visual deviant stimuli containing two red rectangles with 100 ms duration (probability 20%). The stimuli were presented in three 5 min sequences, and each sequence was of 300 stimuli in which the deviant occurred among standards in a pseudorandom fashion, with the constraint that at least two standards were presented between any two deviants. The first 15 stimuli of each sequence were all standard stimuli.

The subjects sat on a comfortable chair in a darkened, sound attenuated, and electrically shielded room. In the center of screen, a black cross was displayed throughout the stimulus blocks. From time to time, the cross became bigger or smaller unpredictably. Participants were instructed to ignore the peripheral stimuli and press the left or the right button as quickly and accurately as possible when the size of the cross became bigger or smaller.

### 2.3. Electroencephalogram Recording and Analysis

The electroencephalogram (EEG) was continuously recorded (bandpass 0.1–200 Hz, sampling rate 1000 Hz) with Neuroscan Scan LT Amplifier, using an elastic cap with 32-channel Ag/Agcl electrodes according to the extended international 10–20 system. The reference electrode was placed on the nose tip. Vertical EOG and horizontal EOG were recorded with two pairs of electrodes, one placed above and below left eye and the other at the right and left lateral canthi. The impedances of the electrodes were maintained below 5 kΩ throughout the experiment. The EOG artifacts were corrected using the correlation method and low-pass filter (30 Hz, 24 dB/octave). The EEG was segmented into the epoch from 100 ms before stimulus to 600 ms after stimulus (100 ms prestimulus period served as a baseline for the amplitude measurement). The trails contaminated with artifacts outside ±100 *μ*V were excluded from averaging. The EEG segments were averaged separately for standard and deviant stimuli in different conditions. MMN was obtained by subtracting ERPs to standard stimuli from ERPs to deviant stimuli for duration. At least 63 deviant stimuli enter the final waveforms.

### 2.4. Statistical Analysis

Statistical analyses were carried out using the software Statistical Package for the Social Sciences, version 13.0 for Windows. According to Figure 2, the most negative peak occurred at 150 to 300 ms after stimulus onset at the occipital-temporal electrode sites. Hence, the mean amplitudes of vMMN were measured for three time windows, including 150–200 ms, 200–250 ms, and 250–300 ms after stimulus onset, respectively. The electrode sites we selected were A1, A2, TP7, TP8, O1, O2, and Oz. To compare the differences of vMMN between two groups, a two-way ANOVA of Group (Hypertension, Normal) × Site (A1, A2, TP7, TP8, O1, O2, Oz) was conducted. The degrees of freedom were corrected using Greenhouse-Geisser's adjustment, and Bonferroni's correction tests were carried out as post hoc analysis. Moreover, the differences in the baseline characteristics were tested by independent-sample *t*-tests; the correlation between the mean amplitude of vMMN and SBP, DBP, and MMSE scores was calculated by one-sample *t*-tests, respectively. A *P* value less than 0.05 was considered statistically significant.

## 3. Results

### 3.1. Characteristics of Study Participants

The mean age of hypertensive patients and normotensives was 53.33 (SD 7.51, age range 43–65 years) years and 52.00 (SD 6.18, age range 43–63 years) years, respectively. The mean level of education was 12.60 (SD 2.64) years for hypertensive participants and 12.27 (SD 2.58) years for controls. As expected, systolic BP and diastolic BP of patients with hypertension were much higher than normal controls (*P* < 0.05). There were no significant differences in the mean of age, sex, education, and MMSE (*P* > 0.05) (Table 1).

### 3.2. Accuracy Rates and Reaction Time

For all participants, mean accuracy of target stimuli was above 94%, showing that participants were paying attention to the cross changes. The mean accuracy rates (ACC) and reaction time (RT) of target stimuli did not differ between patients and normal controls (*P*_*s*_ > 0.05) (Table 2).

### 3.3. ERPs

**Figure 1:**
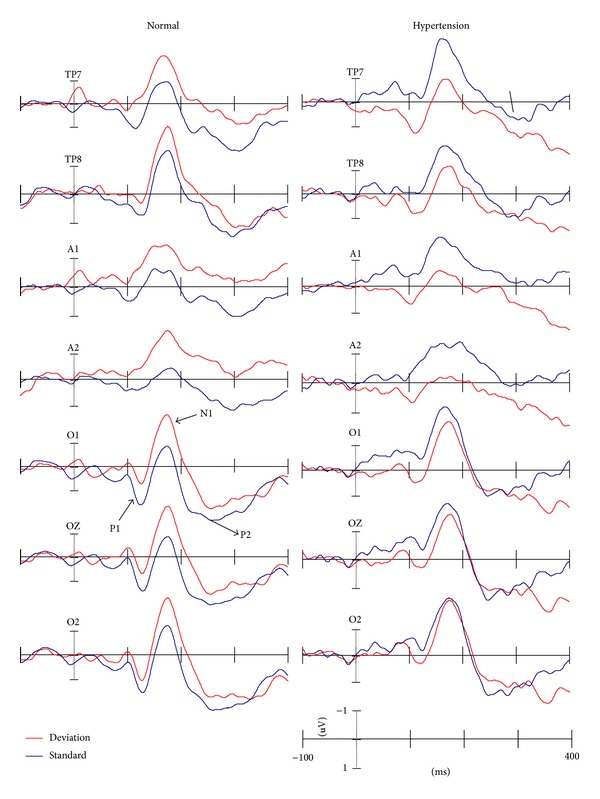
The grand average ERP waveforms at the temporal-occipital sites elicited by deviant and standard stimuli in normal and hypertensive groups, respectively.

**Figure 2:**
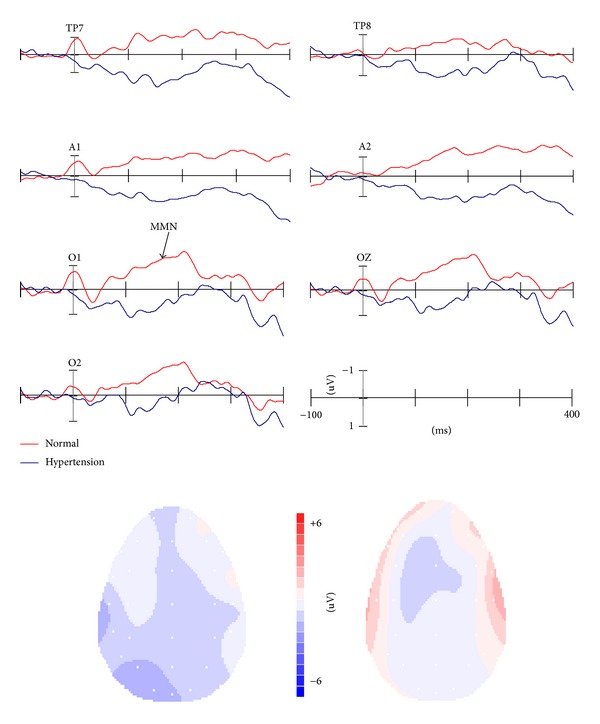
The vMMN elicited by visual duration stimuli at the temporal-occipital sites in normal and hypertensive groups as well as 2D mapping of vMMN in two groups.

Figure 1 showed the grand-averaged ERPs elicited by standard and deviant stimuli at the temporal-occipital electrode sites in both hypertension and control groups. In both groups, P1, N1, and P2 components at the temporal-occipital area were elicited by visual duration stimuli. As shown the difference waveforms in Figure 2, vMMN as the negative detection at the time range of 150–300 ms after stimuli onset was observed. In comparison with controls, the vMMN was decreased in hypertensive patients. Meanwhile, as shown in the 2D scalp distribution of vMMN in Figure 2, the amplitudes of vMMN were larger at the posterior over frontal brain areas. There was a reduction in the temporal-occipital scalp distribution of hypertensive individuals. These differences of latency and amplitude were reported in Tables 3 and 4, respectively.

#### 3.3.1. Peak Latency

The results of two-way repeated ANOVA showed significant main effect of Group on MMN elicited by visual duration (*F*(1, 28) = 6.10, *P* = 0.02, partial *η*2 = 0.18); the peak latency of vMMN was delayed in hypertensive patients (240.8 ms) compared with healthy controls (216.0 ms). Meanwhile, this main effect of Site was more conspicuous at temporal-occipital regions (*F*(1, 28) = 2.31, *P* = 0.04, partial *η*2 = 0.08). However, in both groups the Site × Group interaction was not significant (*F* < 1, *P* > 0.05). The absolute value of latency of vMMN was illustrated in Table 3. Mean Amplitude

For the mean amplitude of vMMN, there was no significant main effect of Group in 200–250 ms time window as well as in 250–300 ms time window. However, during the time range between 150 ms and 200 ms, the vMMN amplitude was significantly smaller (*F*(1, 28) = 6.14, *P* = 0.02, partial *η*2 = 0.18) in the patients with hypertension (0.86 *μ*V) than that in the controls (−1.06 *μ*V). Importantly, the vMMN amplitude of patients had no significant differences from zero (*P* > 0.05). In addition, the main effect of Site was marginally significant (*F*(6, 28) = 1.95, *P* = 0.08, partial *η*2 = 0.07) and post hoc comparisons demonstrated that the vMMN was larger at the occipital electrode sites (O1, O2, and Oz) than left temporal electrode sites (A1, TP7) and right temporal electrode sites (A2, TP8). No other effects were significant (*P* > 0.05) (Table 4).

### 3.4. Correlation between the Mean Amplitudes of vMMN and SBP, DBP, and MMSE Scores

To examine whether the vMMN was related to SBP, DBP, and MMSE of hypertensive individuals, Pearson's correlation analyses were used. The amplitude of the vMMN was not significantly correlated with either SBP or DBP (*P*_*s*_ > 0.05). In addition, there was no significant correlation between MMN and MMSE scores at temporal-occipital sites (*P* > 0.05).

## 4. Discussion

This clinical study suggested that, in the latency range of approximately 150–200 ms after stimuli onset, the peak latency of vMMN was delayed in hypertensive individuals compared with healthy controls. Furthermore, the vMMN amplitude significantly declined in patients with hypertension than that in normal control group. To the best of our knowledge, this is the first report showing impairment of the vMMN in patients with hypertension.

It has been widely proven that the auditory MMN reflects sensory memory-based processing, but the presence of an analogous ERPs' component in the visual modality is still a matter of controversy. Nevertheless, recent vMMN studies summarized that if the paradigm can control visual attention, there is practicability for visual MMN [34]. Therefore, it is crucial to control for conscious attention in the experiment paradigm, designed to argue for a preattentive and automatic sensory information processing in vMMN [24]. In the present study, participants were required to identify the size changes that were cross-successively presented at the center of the screen and to ignore both peripheral stimuli (standard, deviant), which effectively controlled the attention, and this method had been successfully used in previous vMMN experiments [20, 24, 32]. In addition, automatic detection of visual changes was also reflected by a posterior positive component [35, 36], which was considered as an index of detection of a divergent stimulus feature [37]. Whereas we did not access change-related positivity to different durations in our present experiment, possible reasons could be related to different stimuli and contrast of stimuli.

Although no research has investigated the effects of hypertension specifically on preattentive processing, a few previous studies exploring the relation of hypertension and cognitive function have provided some important evidence for this issue. Particularly, many studies have demonstrated that hypertension could result in cognitive impairment [7–11, 28–31]. Goldstein et al. [7] demonstrated that hypertension was associated with faster cognitive decline and a cognitive phenotype characterized by poorer attention and executive functioning and slower processing. Moreover, a cohort study of 1737 rural Chinese elderly people suggested a significant association between hypertension and cognitive decline. This study had the strength of examining cognitive decline using multiple instruments and found that new learning and formation of new memories were significantly associated with hypertension [10]. All these data may suggest changes of preattentive automatically processing as the based stage of information processing. Our study showed that there was no difference between two groups in global cognitive function as assessed by MMSE. Possible reasons could be that MMSE as a measurement of global cognitive function is not sensitive enough and that the study sample was relatively small.

Importantly, some past studies using ERPs' components N2 and P3 measured cognition of hypertensive patients and suggested that information processing and memory are modulated by rise in blood pressure and cognition is delayed in patients with hypertension [30, 31]. Cicconetti et al. [31] recorded ERPs with an odd ball acoustic paradigm to investigate the relationship between SBP levels and neurocognitive function in the early stages of isolated systolic hypertension (ISH). The results showed that the N2 latency of ISH patients was significantly higher than controls (*P* < 0.0001), indicating the existence of early subclinical impairments in neurocognitive function in early ISH, detectable through ERPs. Meanwhile, another study on elderly with borderline isolated systolic hypertension (BISH) by event-related potentials (ERPs) showed that N2 and P300 latencies were significantly higher than the normotensive, which also suggested a gradual change of the cognitive processes related to the increased blood pressure [38].

Our present study verified the damage of preattentive processing in hypertensive subjects. In 150–200 ms time window, the vMMN amplitude of patients with hypertension was significantly smaller, or even absent, compared with controls, as well as the peak latency of vMMN being delayed in hypertensive patients. Although there were no related vMMN studies on patients with hypertension, the changes of MMN elicited by visual duration deviant have been shown in various diseases such as major depressive disorder (MDD) [24]. For instance, in a study comparing the preattentive change detection in patients with a major depressive disorder (MDD) and healthy controls, Qiu et al. [24] found that the mean amplitude of vMMN elicited by exposure duration of visual stimuli was significantly smaller in the patients (−0.91 *μ*V) than in the healthy controls (−2.74 *μ*V). These results suggested functional impairment of preattentive basic visual information processing in MDD patients.

Moreover, we did not find significant correlation between the vMMN amplitude and SBP or DBP, respectively. This result was in line with a previous study of MDD patients; the severity of depressive symptoms had no significant interaction with increment vMMN in MDD patients [24]. Consequently, it was reasonable that the preattentive change detection reflected by MMN component of ERPs was not relevant to the blood pressure level in the hypertensive group. However, the study sample was relatively small, neither the patient's grade nor course of hypertension was considered, and the correlation between the amplitude of vMMN and SBP or DBP needs further study.

Although the results suggested functional impairment of preattentive basic visual information processing in hypertensive patients, the cognitive assessment by MMSE was in the normal range and there was no significant difference between patients and normal controls. Moreover, there was no correlation between the mean amplitudes of vMMN and MMSE in patients with hypertension. Consistent with our finding, Cicconetti et al. [31] obtained similar results during N2 and P300 ERPs study in patients with ISH. Therefore, these findings suggest the detectable existence of early subclinical alterations in preattentive change detection in hypertensives through vMMN component of ERPs. In our study, we first took event-related potentials (ERPs) by recording the mismatch negativity (MMN) indicating dysfunction of preattentive processing in patients with hypertension. However, the patient's age and grade and course of hypertension were not considered, which may contribute different effects to preattentive processing.

## 5. Conclusions

In conclusion, our study provides evidence suggesting the impairment of preattentive processing in hypertensive patients. Besides, the impairment of preattentive processing could be a distinguishing trait of hypertension but unrelated to the blood pressure levels. Moreover, the decreased amplitude of MMN was more sensitive and reliable than MMSE to assess cognitive impairment of patients with hypertension. Findings from this study have significant implications for better understanding the neural mechanisms that link hypertension to cognitive impairment, dementia, and Alzheimer's disease. Future studies will be necessary to determine whether the grade or course of hypertensive patients affects preattentive processing.

## Figures and Tables

**Table 1:** The baseline characteristics of the participants.

Baseline characteristics	Hypertensive (*n* = 15)	Normotensive (*n* = 15)	*P* value
Number of males	8	8	
Age (years), mean (SD)	53.33 (7.51)	52.00 (6.18)	0.60
Education (years), mean (SD)	12.60 (2.64)	12.27 (2.58)	0.73
Systolic BP (mmHg), mean (SD)	168.73 (14.07)	123.87 (9.88)	0.00
Diastolic BP (mmHg), mean (SD)	90.47 (9.16)	73.93 (6.63)	0.00
MMSE scores, mean (SD)	27.53 (1.36)	28.07 (1.28)	0.28

*P* value is for test of difference between hypertensive and normotensive.

BP: blood pressure; SD: standard deviation.

**Table 2:** Mean and standard deviation of accuracy rates and reaction times for the hypertensive patients and normal controls.

	Hypertensive patients	Normal controls	*P* value
RT (ms), mean (SD)	330.22 (49.02)	335.22 (30.57)	0.74
ACC (%), mean (SD)	98.56 (2.62)	99.24 (1.78)	0.41

*P* value is for test of difference between hypertensive patients and normal controls.

SD: standard deviation; ACC: accuracy rates; RT: reaction times.

**Table 3:** The vMMN latencies elicited by duration deviant in patients with hypertension and normal controls.

Latency (ms)	Hypertensive patients	Normal controls	*P* value
TP7	263.4 (11.1)	232.1 (11.1)	0.06
TP8	242.1 (12.8)	211.5 (12.8)	0.10
A1	244.9 (11.8)	220.1 (11.8)	0.15
A2	240.1 (13.2)	224.5 (13.2)	0.41
O1	237.7 (9.8)	196.6 (9.8)	0.006
OZ	231.9 (10.7)	216.5 (10.7)	0.32
O2	225.5 (10.6)	210.7 (10.6)	0.33

Total	240.8 (7.1)	216.0 (7.5)	0.02

*P* value is for test of difference between hypertensive patients and normal controls.

Data were expressed as mean (standard error).

**Table 4:** The vMMN amplitudes elicited by duration deviant in patients with hypertension and normal controls.

Amplitude (*μ*V)	Hypertensive patients	Normal controls	*P* value
TP7	1.5 (0.7)	−1.0 (0.7)	0.02
TP8	0.9 (0.6)	−0.7 (0.6)	0.07
A1	1.2 (0.7)	−0.9 (0.7)	0.04
A2	1.1 (0.6)	−1.3 (0.6)	0.004
O1	0.6 (0.6)	−1.3 (0.6)	0.03
OZ	0.5 (0.5)	−1.2 (0.5)	0.04
O2	0.2 (0.6)	−1.1 (0.6)	0.15

Total	0.9 (0.5)	−1.1 (0.5)	0.02

*P* value is for test of difference between hypertensive patients and normal controls.

Data were expressed as mean (standard error).
